# What is thyroid function in your just-diagnosed cancer patient?

**DOI:** 10.3389/fendo.2023.1109528

**Published:** 2023-02-17

**Authors:** Aleck Hercbergs, Shaker A. Mousa, Hung-Yun Lin, Paul J. Davis

**Affiliations:** ^1^ Department of Radiation Oncology, The Cleveland Clinic, Cleveland, OH, United States; ^2^ Albany College of Pharmacy and Health Sciences, Albany, NY, United States; ^3^ PhD Program for Cancer Molecular Biology and Drug Discovery, College of Medical Science and Technology, Taipei Medical University, Taipei, Taiwan; ^4^ Department of Medicine, Albany Medical College, Albany, NY, United States

**Keywords:** L-thyroxine (T4), 3,5,3’-triiodo-L-thyronine (T3), reverse T3 (rT3), cancer, metastasis, euthyroid hypothyroxinemia, thyroid function tests

## Abstract

The principal hormonal product of the thyroid gland, L-thyroxine (T4), is a prohormone for 3,3’,5-triiodo-L-thyronine, T3, the major ligand of nuclear thyroid hormone receptors (TRs). At a cell surface thyroid hormone analogue receptor on cancer cell and endothelial cell plasma membrane integrin αvβ3, however, T4 at physiological concentrations is biologically active and is the major ligand. At this site in solid tumor cells, T4 nongenomically initiates cell proliferation, is anti-apoptotic by multiple mechanisms, supports radioresistance and enhances cancer-related angiogenesis. In contrast, hypothyroidism has been reported clinically to slow tumor growth. At physiological levels, T3 is not biologically active at the integrin and maintenance of euthyroidism with T3 in cancer patients may be associated with slowed tumor proliferation. Against this background, we raise the possibility that host serum T4 levels that are spontaneously in the upper tertile or quartile of the normal range in cancer patients may be a factor that contributes to aggressive tumor behavior. Recent observations on tumor metastasis and tumor-associated propensity for thrombosis due to T4 also justify clinical statistical analysis for a relationship to upper tertile hormone levels. That reverse T3 (rT3) may stimulate tumor growth has recently been reported and thus the utility of adding this measurement to thyroid function testing in cancer patients requires assessment. In summary, T4 at physiological concentrations promotes tumor cell division and aggressiveness and euthyroid hypothyroxinemia arrests clinically advanced solid tumors. These findings support the clinical possibility that T4 levels in the upper tertile of the normal range require examination as a tumor supporting factor.

## Introduction

The description of a thyroid hormone analogue receptor on plasma membrane integrin αvβ3 (1) has afforded new insights into how thyroid hormone works. For example, the principal ligand of the integrin receptor is L-thyroxine (T4), a hormone analogue viewed almost exclusively as the prohormone for 3,5,3’-triiodo-L-thyronine (T3), the form of the hormone which is the ligand for the essential nuclear receptors (TRs) for thyroid hormone (2, 3). In addition, 3,3’,5’-triiodo-L-thyronine (reverse T3, rT3) is also a ligand at physiological pH of the integrin receptor for T4, but is generally viewed as function-free in the nuclear compartment (4).

The thyrointegrin receptor is highly functional in cancer cells and rapidly-dividing endothelial cells. In normal, nonmalignant cells, however, integrin αvβ3 is underexpressed and is not in an activated, i.e., extended conformation, state (3). Signals generated at the integrin thyroid hormone analogue receptor on the cancer cell surface are converted into changes in specific gene expression by a variety of signal transduction that in large measure are independent of TRs (3, 5, 6). These effects are achieved at physiological levels of T4 and cause us to raise the possibility here that T4 concentrations in the upper tertile or quartile of the normal range may be tumor supporting, compared to lower tertile values.

## Reported contributions of thyroid hormone to cancer cell functions

### Biological activity of T4 in cancer cells

*In vitro* and *in vivo* preclinical observations and population-based clinical studies document cancer-stimulating effects of thyroid hormone, as discussed below. Abundant chemical, clinical and epidemiological evidence implicates L-T4 in solid tumor genesis, growth and mortality. The putative mechanism involves the thyroid hormone analogue receptor on the plasma membrane integrin αvβ3 expressed by proliferating cancer cells and vascular endothelium, but not in normal, untransformed cells. Preclinical genomic evidence that the presence of T4 at cancer cell integrin αvβ3 is anti-apoptotic and pro-angiogenic is included in **Table 1**. Systemic depletion of T4 in the clinical setting of cancer may contribute to decreased tumor volume (15).

**Table 1:** Genes relevant to angiogenesis and to apoptosis whose expression is differentially regulated nongenomically by T4 at integrin αvβ3 on tumor cells.

Gene	Up- or down-regulation of Transcription	Reference
Pro-angiogenesis genes		
*FGF2*	Incr	(7)
*HIF*-1*a*	Incr	(8)
*INOS*	Incr	(9)
*MMP*-*9*	Incr	(10)
*VEGF*	Incr	(11)
Pro-apoptosis genes		
*APAF*-*1*	Decr	(12)
*CASP3*	Decr	(12)
*NOXA*	Decr	(12)
*PUMA*	Decr	(12)
Anti-apoptosis genes		
*COX*-*2*	Incr	(8)
*SREBP-1*	Incr	(13)
*XIAP*	Incr	(14)

APAF-1, apoptosis protein activating factor 1; CASP3, caspase-3; COX-2, cyclooxygenase-2; FGF2, fibroblast growth factor 2; HIF-1α, hypoxia-inducible factor-1; INOS, inducible nitric oxide synthase; MMP-9, matrix metalloproteinase-9; NOXA = PMAIP1, phorbol 12-myristate 13-acetate-induced protein 1; PUMA, p53 upregulated modulator of apoptosis; SREBP-1, sterol regulatory element-binding protein 1; VEGF, vascular endothelial growth factor; XIAP, X-linked inhibitor of apoptosis.

Incr, increased expression; Decr, decreased expression [From Davis et al. (3)].

The anti-apoptotic genomic activity of T4 may reflect increased hormone-induced expression of anti-apoptotic genes or decreased expression of pro-apoptotic genes (**Table 1**). The complex and comprehensive anti-apoptotic actions of T4 are integrated into the extrinsic (Fas receptor) and intrinsic (hypoxia-oxidative stress) pathways of apoptosis in tumor cells (3). The pathways converge at the mitochondrion and the actions of T4 would appear to protect mitochondrial function in cancer cells challenged by pro-apoptotic stresses that could include chemotherapy.

**Figure 1:**
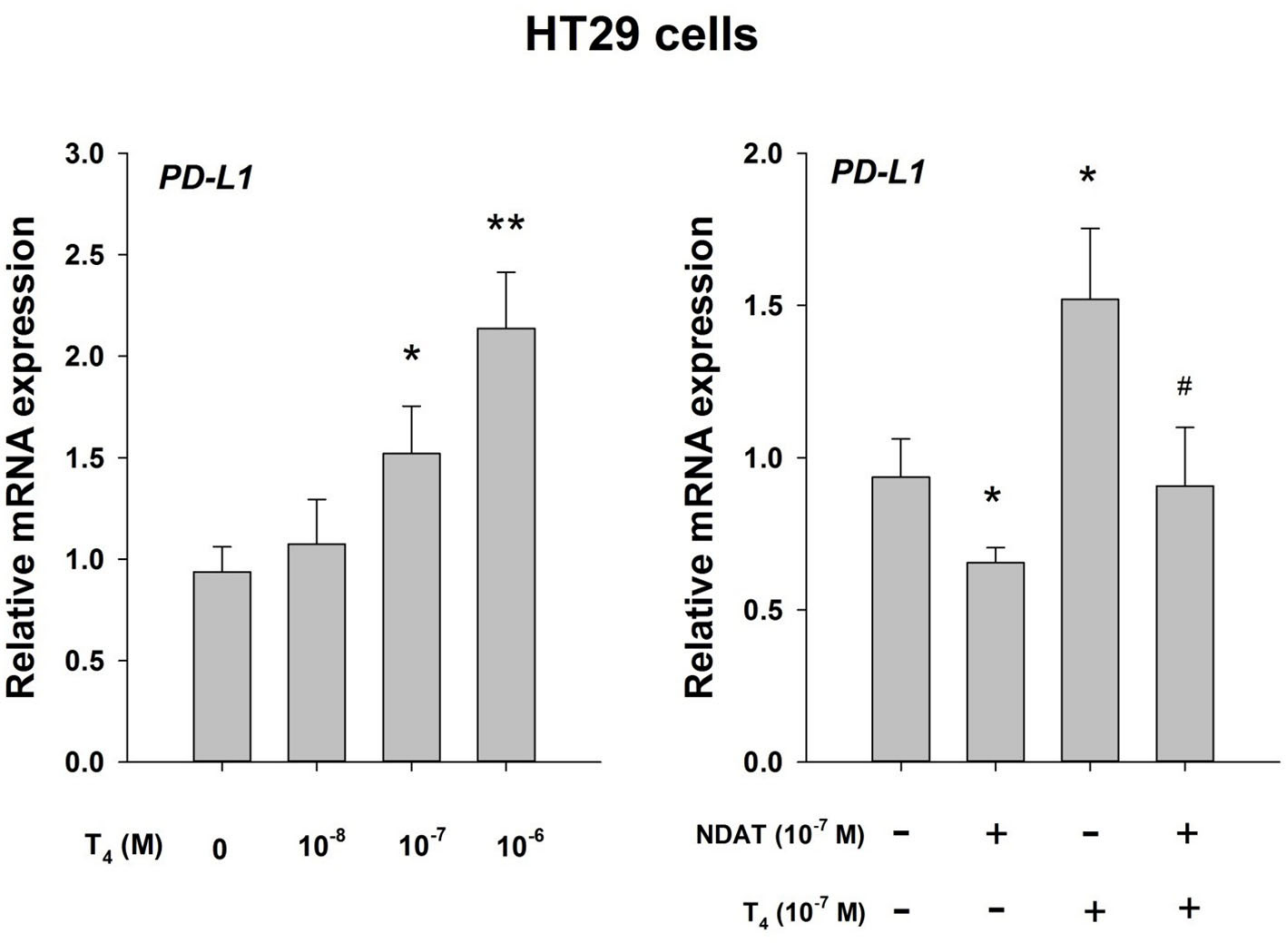
Thyroid hormone as T4 inhibits host anticancer immune response *via* enhanced PD-L1 gene expression. Effect of T4 and the T4 derivative, NDAT (nano-diamino-tetrac), on induction of PD-L1 mRNA in HT-29 human colorectal cancer cells. Hormonal effects are expressed relative to control samples that lacked T4. Left panel, T4 concentration-dependence of PD-L1 mRNA expression with exposure of cells to hormone for 24 h. Right panel, the action of T4 on induction of PD-L1 mRNA is inhbited by NDAT, a specific inhibitor of T4 action at its receptor site on tumor cell surface integrin αvβ3 (3). Reproduced by permission *p < 0.05, **p < 0.01, compared with control; #p < 0.05, compared with T4 at 10-7 M. from HY Lin et al. (16).

T4 is active at the PD (programmed death)-1/PD-L(ligand)1 immune checkpoint (14, 16) (**Figure 1**). This effect is pharmacologic at integrin αvβ3 and does not involve the immune system. The T4 effect is subject to pharmacologic inhibition by a chemically modified T4 derivative (tetraiodothyroacetic acid, tetrac)that has a variety of antitumor actions (3, 5, 6). We propose that endogenous circulating T4 is capable of opposing immunotherapy of cancer directed at the PD-1/PD-L1 immune checkpoint. Tetrac-based agents directed at the thyroid hormone binding site on integrin αvβ3 conceptually is adjunctive.

T4 depletion may also enhance tumor cell killing by ionizing radiation (17) and by chemotherapeutic agents (18). We have noted above that euthyroid hypothyroxinemia and TKI-induced hypothyroidism have favorable clinical impacts. What is some of the evidence that a spontaneously low normal vs. high normal free T4 level impact tumor outcome?

The prospective Rotterdam population study of cancer risk among people not known to have thyroid disease revealed that a downward trend in circulating thyrotropin (TSH) levels measured years prior to diagnosis of cancer and suggestive of hyperthyroid function were associated with an overall increased risk of cancers and, specifically, of higher risk of lung and prostate cancers (19). Increased upper tertile levels of FT4 were associated with a higher risk of solid cancers (HR, 1.42; 95% confidence interval [CI], 1.12-1.79) and specifically of lung cancer (HR, 2.33; 95% CI, 1.39-3.92) and breast cancer (HR 1.77; 95% CI, 1.10-2.84). The risk estimates were similar after exclusion of thyroid hormone-altering medications, but the breast cancer observations lost significance. Compared with the lower FT4 tertile, the highest tertile was associated with a 1.13-fold increased risk of any solid tumor, 1.79-fold increased risk of lung cancer and a 1.14-fold increased breast cancer risk (P <0.05 for all trends).

A study by M. Cristofanilli et al. (20) at MD Anderson Cancer Center examined the apparent impact of spontaneous hypothyroidism on the risk of developing breast cancer and on the prognosis of established breast cancer. Hypothyroidism was significantly less common in breast cancer patients than controls (7% vs. 14.9%, providing an odds ratio of 0.43 for primary hypothyroidism in breast cancer patient versus controls.

Finally, in a prospective study of more than 29,000 people without prior histories of thyroid disease, Hellevik et al. (21) measured baseline serum TSH and determined cancer incidence over a follow-up period of 9 years. Low thyrotropin levels (<0.50 mU/L) were associated with increased cancer risk (adjusted hazard ratio [HR], 1.34; 95% CI, 1.06-1.69), compared with the euthyroid reference group. The higher risk was driven by lung cancer (adjusted HR, 2.34; 95% CI, 1.24-4.40) and prostate cancer (adjusted HR, 1.97; 95% CI, 1.04-3.76). After excluding the first two years of follow-up, the associations between low TSH and cancer risk were strengthened for lung cancer to 2.9 (1.49-5.70) and for prostate cancer to 2.60 (1.36-4.99).

These cancer data indicate that high normal (upper tertile) circulating thyroid hormone levels and downward trend in TSH within the normal range are associated with increased cancer risk. Further support for this risk is that interventional hypothyroxinemia (15) in patients with advanced solid tumors is associated with tumor regression and improved survival. The mechanism is thought to involve decreased action of T4 specifically at the plasma membrane receptor for thyroid hormone analogues.

### Activity of reverse T3 in cancer cells

Reverse T3 is the third most common iodothyronine in the human circulation (22). It is generated from T4 by cellular deiodinase 3 (DI03, D3) or D1 and removal of the 5’ iodine (4). Because of its lack of genomic (nuclear receptor-dependent) activity, rT3 has been thought to be of little or no physiological or pathophysiological importance (2, 3). However, rT3 is now appreciated to have nongenomic effects, e.g., conversion of soluble actin to fibrous (F) actin (23) and stimulation at physiological rT3 concentrations of proliferation of cancer cells (4). Such proliferation of human breast cancer and glioblastoma cells induced *via* the thyroid hormone receptor on integrin αvβ3 may be increased by 50% or more within 24 hours. These preclinical studies need clinical evaluation of possible correlations of circulating rT3 levels and cancer growth. The relevance of increased circulating rT3 to cancer biology arises from the nonthyroidal illness syndrome (NTIS) (24) and altered deiodination that accompanies cancers and other systemic illnesses; circulating levels of T3 decrease in NTIS and rT3 levels are often increased in this syndrome.

It is also important to appreciate that control of cancer cell abundance of F actin by factors such as rT3 may be of substantial importance to cancer cell invasiveness and metastasis.

### Biological activity of T3 in cancer cells

In preclinical studies, supraphysiologic concentrations of T3 are required at the thyroid hormone analogue receptor on integrin αvβ3 in cells from various types of solid tumors to reproduce actions of T4 at physiological concentrations on cell proliferation, migration and tumor-linked angiogenesis (2, 3). In a clinical study of advanced cancers, pharmacologic elimination of host T4 production by the thyroid gland and maintenance of euthyroidism with T3 (euthyroid hypothyroxinemia) was associated with arrest of tumor growth or reduction in tumor size (15). Thus, there is a divergence that appears to be exploitable in solid tumor cells between the predominantly metabolic (with little or no oncogenic potential) actions of T3 and the pro-oncogenic activity of T4. In contrast to cells from solid tumors, cells from certain hematological malignancies may proliferate in response to physiological levels of T3 (12, 25).

### Crossing by T4 of the blood-brain barrier in the settings of GBM, metastases from other organs

The transport of T4 across the blood-brain barrier is essential to normal brain development *in utero* and childhood (26–28). Among the factors important to this transport is the serum thyroid hormone transport protein, transthyretin (TTR) or thyroid hormone-binding prealbumin (TBPA). The development of primary cancers of the brain, e.g., glioblastoma, and of cancer metastases may also have some dependence on the availability of T4 (29). The passage of T4 bound to TTR proceeds through the choroid plexus and into cerebrospinal fluid (CSF). Chemically-modified tetraiodothyroacetic acid (tetrac), a derivative of T4, has been shown to concentrate highly in tumors xenografted into mouse brain (30), consistent with the drug’s presentation *via* TTR to the blood-brain barrier transport system.

### Clinical findings in patients in whom it is proposed that thyroid function should be defined

Our contention here is that all patients with a diagnosis of cancer deserve thyroid function-testing and ranking according to tertile of the normal range. But are there specific clinical findings in cancer patients that particularly raise the possibility that thyroid hormone analogues in the upper tertile of the normal range are possibly contributing to the clinical profile?

*Tumor metastasis.* We have shown that T4 can support cancer metastasis by multiple mechanisms (29). These include increased cell expression of metalloproteinase genes, angiogenesis-relevant genes, the epithelial-mesenchymal transition (EMT) process and promotion of cancer cell-platelet interactions. Substantial clinical evidence supports the contribution of the normal thyroid function state and T4 to the metastatic process of solid tumors (15, 20, 31, 32). The cell surface thyrointegrin receptor site for thyroid hormone analogues mediates this action of T4 (3).

There is clinical evidence that hypothyroidism may sometimes support development of metastases by certain cancers (33–35). The mechanism could be loss of thyroid hormone actions on the immune system that depend upon nuclear receptors for T3 (36).

*Rapid primary tumor growth.* T4 is a stimulatory factor for tumor cell division, angiogenesis and support of anti-apoptosis mechanisms (3).

*Growth of more than one primary tumor in an individual patient.* That a proliferative contribution of upper tercile levels of host T4 may exist to genetically condition multiple primary cancer syndromes, such as multiple endocrine neopl.asia (37) and Caney Complex (CNC) (38), requires investigation. The existence of active T4 receptors on integrin αvβ3 of cells of many types of solid tumors underlies T4-induced proliferation in such cells (3) and evidence that pharmacologic elimination of host T4 reduces tumor growth (15).

*Emergence of chemoresistance and/or radioresistance in tumors without cancer cell gene mutations known to be associated with –resistance syndromes.* The control of the conformational state by T4 is a factor that contributes to radioresistance (17). Among the mechanisms by which T4 supports chemoresistance is stimulation of the tumor cell plasma membrane P-glycoprotein (P-gp) efflux pump (18). T4 may also stimulate PD-1 and PDL1 gene expression in cancer cells, thus raising the possibility of specific resistance to immune checkpoint inhibitor agents. T4 has also been shown to enhance expression of VEGFR and EGFR genes (3), actions which may reduce effectiveness of anti-VEGFR and -EGFR anticancer drugs.

*Cancer-associated thrombosis*. T4 promotes platelet adherence (39), as mentioned above. This effects may enhance the actions of other thrombosis-promoting mechanisms.

We propose that, given its anticancer activity, endogenous, naturally occurring tetrac levels should be compared serially in healthy subjects and cancer-bearing patients to begin to address the question of whether the hormone analogue confers some degree of cancer protection or, if low levels of already modest concentrations of tetrac are associated with cancer risk.

## Discussion

Observations cited here raise the possibility that T4 and, possibly, rT3, have supportive roles in cancer cells. A large number of reports have shown that, indeed, T4 and rT3 at physiological concentrations promote cancer cell division in a variety of model systems. At physiological concentrations, systems. We have reported that T3 is not a growth factor for tumor cells. Stimulatory actions of T3 on cancer cells reported by others appear to have depended upon elevated T3 concentrations (40, 41). As noted above, T4 may also support radioresistance of cancer cells, serve as an anti-apoptotic agent and also stimulate tumor-linked local angiogenesis (3). In cancer patients, we have shown that pharmacological elimination of T4 and maintenance of eumetabolism with exogenous T3 (euthyroid hypothyroxinemia) may substantially reduce solid tumor growth (15). Reduced levels of circulating thyroid hormone (hypothyroidism) have been shown by other investigative groups to slow cancer growth (42, 43). The anti-cancer properties of tyrosine kinase inhibitor (TKI) anticancer agents may depend in patients with renal cell carcinoma to be a function of the side effect of TKIs to induce hypothyroidism (32).

Are there are other thyroid hormone analogues to consider as modifiers of tumor cell biology? Tetrac is a naturally occurring deaminated derivative of T4 (3, 44). It is seen to be of low biologic thyroid hormone activity (44) as a T4-like agent in terms of nuclear receptor-mediated effects, but a large number of reports have documented in preclinical studies its anticancer activity mediated by the thyroid hormone analogue receptor on cancer cell plasma membrane integrin αvβ3 (3). It has anti-angiogenic activity that is mediated by the integrin in rapidly-dividing endothelial cells (3). Endogenous tetrac levels tend to correlate in normal subjects with circulating total T4 and free T4 (FT4) (45), but may decrease in acute and subacute systemic illnesses (46). Tetrac has not been studied as a possible component of NTIS.

We propose that naturally occurring levels of tetrac—given the latter’s anticancer activity—should be compared serially in healthy subjects and cancer-bearing patients to begin to address the question of whether the hormone analogue confers some degree of cancer protection or, if low levels of already modest concentrations of tetrac are associated with cancer risk.

In conclusion, we propose that the state of thyroid function may be a factor regulating the rate of solid tumor growth in cancer patients. ‘State of thyroid function’ may simply be endogenous regulation of pituitary-thyroid gland function or in patients with a history of hypothyroidism and T4 replacement therapy may reflect T4 dosing. In patients with a history of TSH suppression for thyroid gland tumors, relatively high dosage of exogenous T4 is justified. We suggest here that circulating endogenous levels of T4 in the upper tertile or quartile of the normal range requires investigation in cancer patients is a factor possibly contributing to tumor growth. The possibility that TSH-suppressed patients on high-dose T4 may be subject to development of nonthyroid cancers can initially be examined in retrospective analyses.

## Data availability statement

The original contributions presented in the study are included in the article/supplementary material. Further inquiries can be directed to the corresponding author.

## Author contributions

PD wrote the first draft of the manuscript. AH, SM, and H-YL edited the manuscript and have made major research contributions to our understanding of the tumor-support activity of L-thyroxine.P. All authors contributed to the article and approved the submitted version.
